# Prevalence and breed predisposition for spinal diseases in pugs and French bulldogs

**DOI:** 10.1002/vetr.5122

**Published:** 2025-01-23

**Authors:** Stephanie Kerr, Eleftheria Skovola, Lowri Maskell, Steven De Decker

**Affiliations:** ^1^ Department of Clinical Science and Services Royal Veterinary College Hatfield UK; ^2^ Department of Neurology Dick White Referrals Six Mile Bottom UK

## Abstract

**Background:**

Pugs and French bulldogs (FBDs) are commonly presented for spinal disease. The aim of this study was to define the most common spinal diseases in both breeds.

**Methods:**

This was a monocentric retrospective study including pugs and FBDs presenting with clinical signs consistent with spinal disease between 2010 and 2022. The information collected included signalment, onset of clinical signs, presence of spinal pain, progression of clinical signs, presence of urinary or faecal incontinence and neurological examination findings.

**Results:**

A total of 439 FBDs with 448 diagnoses and 106 pugs with 125 diagnoses were included in the study. A total of 272 (62.0%) FBDs had an acute onset of clinical signs, and 79 pugs (74.5%) had a chronic onset. The most common disease in the FBD population was acute intervertebral disc extrusion, with 377 diagnoses (84.3%). The most common diagnoses among pugs were spinal arachnoid diverticula (SADs) (*n* = 30, 24.0%) and chronic intervertebral disc protrusion (*n* = 30, 24.0%).

**Limitations:**

This study was limited by its retrospective nature, meaning that there was not a standardised diagnostic protocol.

**Conclusion:**

This study confirms that FBDs and pugs are affected by different spinal disorders. While FBDs are mostly affected by acute intervertebral disc extrusions, pugs are more often affected by SADs and chronic intervertebral disc protrusions.

## INTRODUCTION

Pugs and French bulldogs (FBDs) have a similar phenotype, both being small‐breed brachycephalic dogs. As a result of their phenotypic similarities, they are predisposed to many of the same conditions,^1^ including brachycephalic obstructive airway syndrome^2^ and ocular disease.^3^ With the rising popularity of these brachycephalic breeds, there is increasing evidence that they are also at risk from multiple spinal diseases, including spinal arachnoid diverticula (SAD),^4^, ^5^, ^6^, ^7^, ^8^ intervertebral disc disease^4^, ^9^ and congenital vertebral malformations.^10^, ^11^, ^12^ However, it is currently unclear what factors may lead to both breeds being commonly presented for spinal disease and despite their similarities, existing studies have suggested important differences between them.^10^, ^11^, ^13^, ^14^, ^15^, ^16^, ^17^

Both breeds have a high prevalence of congenital vertebral malformations, including hemivertebrae and caudal articular process dysplasia (CAPD).^10^, ^11^, ^15^, ^16^ One study revealed that 93.5% of neurologically normal FBDs and 17.6% of neurologically normal pugs have at least one thoracic hemivertebrae detected via diagnostic imaging studies.^10^ Interestingly, it has been suggested that thoracic hemivertebrae will only rarely result in clinical signs in FBDs, while it seems to be of greater clinical relevance in pugs.^10^, ^13^, ^15^ CAPD, characterised by aplasia or hypoplasia of one or more caudal articular processes along the vertebral column, has strongly been associated with the pug breed.^18^ The clinical relevance of this vertebral malformation has, however, been debated, with one study demonstrating that 97.0% of neurologically normal pugs and 70.4% of neurologically normal FBDs have at least one site of CAPD along their vertebral column.^11^ Although it has been speculated that CAPD can result in meningeal fibrosis, meningeal fibrosis has also been demonstrated in pugs without vertebral malformations.^19^ The relationship between both conditions therefore remains unclear.^19^ Other studies have also failed to identify a clear association between CAPD and the development of other spinal conditions, such as intervertebral disc disease and SAD.^20^, ^21^, ^22^

Despite the almost ubiquitous nature of vertebral body malformations in FBDs, intervertebral disc disease is by far the most common spinal condition in this breed.^4^ One study suggested that 70.3% of FBDs with spinal disease suffer from cervical or thoracolumbar intervertebral disc extrusions, followed by great distance by SADs as the second most common spinal disorder.^4^

Despite the phenotypic similarities, such information is currently not available for pugs. As we increase our knowledge of spinal disorders, the importance of recognising the two breeds separately when it comes to considering the prevalence of spinal diagnoses becomes more evident. By identifying the most likely differential diagnoses based on signalment and clinical presentation, it might be possible to prioritise diagnostic steps and discuss likely outcomes, treatment options and prognoses with the owner. Despite it being apparent that these dogs suffer from a multitude of spinal conditions, no study has yet compared the prevalence and clinical presentation of spinal disorders between FBDs and pugs.

The aim of this study was to define the most common spinal diseases in both pugs and FBDs. These two breeds were chosen for comparison due to their similar conformations, which differ vastly from that of the dachshund–the breed most predisposed to acute intervertebral disc extrusions.^23^ Their phenotypic similarities are already well recognised and are known to lead to their overrepresentation for upper airway and ocular disease^2^ but could perhaps also explain the high prevalence at which both breeds present for spinal disease. Although it is currently unknown if pugs suffer from a similar distribution of spinal conditions to FBDs, we hypothesise that pugs are less commonly affected by acute intervertebral disc disease and more commonly by chronic and complex spinal conditions. Therefore, we hypothesise that FBDs are most commonly affected by acute intervertebral disc extrusions, whereas pugs are more often diagnosed with chronic spinal diseases, including SAD, chronic intervertebral disc protrusions, constrictive myelopathy and hemivertebra with kyphosis.

## MATERIALS AND METHODS

The digital medical database of the Queen Mother Hospital for Animals at the Royal Veterinary College (RVC) was reviewed retrospectively to identify pugs and FBDs presented with spinal disease between January 2010 and March 2022. The database was searched using terms, including ‘myelopathy’, ‘cervical hyperaesthesia’, ‘thoracic hyperaesthesia’, ‘lumbar hyperaesthesia’, ‘C1‒C5’, ‘C6‒T2’, ‘T3‒L3’ and ‘L4‒S3’. Requirements for inclusion in this study included having full medical records and a diagnosis of spinal disease based on advanced imaging. If dogs did not have full medical records, a definitive final diagnosis was not reached or their imaging studies were not available, then they were excluded. Patients were excluded if their clinical signs were not a consequence of a primary spinal disorder (e.g., syringomyelia secondary to Chiari‐like malformation). Patients were excluded if they had more than one clinically relevant condition at separate spinal cord segments. The information collected included signalment, onset of clinical signs, presence of spinal pain, progression of clinical signs, presence of urinary or faecal incontinence and neurological examination findings.

All dogs in the study had their diagnoses reached using advanced imaging with high‐field magnetic resonance imaging (MRI) under general anaesthesia (1.5 T; Intera; Philips Medical Systems) with or without computed tomography (CT; 16 or 320 slice CT scanner). MRI studies included a minimum of T1 and T2‐weighted sagittal and transverse sequences. Standard image archiving and communication system software (eUnity v7.1.1, www.eunity.rvc.ac.uk) was used to evaluate all imaging studies. In all cases, images were reviewed at the time of diagnosis by a board‐certified radiologist and neurologist. In patients with inflammatory CNS disease, the diagnosis was reached with a combination of MRI findings and compatible cerebrospinal fluid analysis results. Constrictive myelopathy was diagnosed based on imaging characteristics described in the 2020 study by Lourinho et al.,^24^ including a V‐shaped extradural lesion, intramedullary changes and an irregular subarachnoid space. Acute intervertebral disc extrusions are defined as herniation of degenerate nucleus pulposus.^25^ They were diagnosed according to imaging features, including lateralised extradural material, completely degenerate intervertebral disc and extruded material extending beyond the area of the intervertebral disc.^26^, ^27^ Conversely, chronic intervertebral disc protrusions are defined as dorsal bulging of the annulus fibrosus and consequently the dorsal longitudinal ligament into the spinal canal.^25^ They were diagnosed according to imaging features, including midline herniation and only partially degenerate nucleus pulposus.^26^, ^27^

The onset of clinical signs was defined as peracute (<2 days), acute (2‒7 days), subacute (7‒14 days) or chronic (>14 days) according to the time to presentation. CAPD was not included as a separate disease entity in this study due to the almost omnipresent nature of this malformation in neurologically normal pugs, the very high prevalence in neurologically normal FBDs^11^ and the uncertain clinical relevance of this vertebral anomaly.

For dogs with thoracolumbar lesions, the severity of neurological signs was graded from 0 to 5 using the modified Frankel score,^28^ which was defined as paraplegia with no deep nociception (grade 0), paraplegia with no superficial nociception (grade 1), paraplegia with intact nociception (grade 2), non‐ambulatory paraparesis (grade 3), ambulatory paraparesis (grade 4) and spinal hyperaesthesia only (grade 5). For dogs with cervical lesions, the severity of neurological signs was graded using a system adapted from Taylor‐Brown et al.^29^ They were graded from 0 to 4, which was defined as tetraplegia with respiratory compromise (grade 0), tetraplegia without respiratory compromise (grade 1), non‐ambulatory tetraparesis (grade 2), ambulatory tetraparesis (grade 3) and cervical hyperaesthesia without a gait abnormality (grade 4).

The data were analysed using a commercially available statistical software package (SPSS, version 29). Data were assessed for normality, and a Mann‒Whitney *U*‐test was used to analyse non‐parametric data. The chi‐squared test was used to determine the association between breed and parametric variables. Post hoc analysis was performed using a Bonferroni test, excluding diagnoses with less than five cases. *p*‐Values of less than 0.05 were considered significant for all data.

## RESULTS

A total of 474 FBDs and 117 pugs were presented at the Queen Mother Hospital for Animals with clinical signs consistent with spinal disease within the defined study period. Of these, 26 FBDs and 11 pugs were excluded from the study due to incomplete clinical records, lack of primary spinal disease, lack of advanced imaging or having more than one clinically relevant condition at separate spinal cord segments. Following exclusion of the cases that did not meet the inclusion criteria, 439 FBDs with a total of 448 diagnoses and 106 pugs with a total of 125 diagnoses were included.

The diagnoses reached for both FBDs and pugs (Table 1) included acute intervertebral disc extrusion (*n* = 390), SAD (*n* = 52), chronic intervertebral disc protrusion (*n* = 37), hemivertebrae with kyphosis (*n* = 33), inflammatory CNS disease (*n* = 17), constrictive myelopathy (*n* = 15), acute herniation of non‐degenerate nucleus pulposus (*n* = 8), discospondylitis (*n* = 7), ischaemic myelopathy (*n* = 5), thoracic vertebral canal stenosis associated with vertebral arch anomalies (*n* = 3), vertebral body osteomyelitis (*n* = 2), spinal epidural empyema (*n* = 1) and spinal dysraphism (*n* = 1).

**TABLE 1:** Distribution of diagnoses of spinal disease in French bulldogs and pugs

Diagnosis	Number (percentage of total) in pugs		Number (percentage of total) in French bulldogs	
	Cervical	Thoracolumbar	Cervical	Thoracolumbar
Spinal arachnoid diverticula	5 (4.0%)	25 (20.0%)	1 (0.2%)	21 (4.7%)
Chronic intervertebral disc protrusion	5 (4.0%)	25 (20.0%)	4 (0.9%)	3 (0.7%)
Hemivertebrae with kyphosis	0 (0.0%)	21 (16.8%)	0 (0.0%)	13 (2.9%)
Constrictive myelopathy	0 (0.0%)	15 (12.0%)	0 (0.0%)	1 (0.2%)
Acute intervertebral disc extrusion	5 (4.0%)	8 (6.4%)	125 (27.9%)	252 (56.3%)
Acute herniation of non‐degenerate nucleus pulposus	4 (3.2%)	3 (2.4%)	0 (0.0%)	1 (0.2%)
Inflammatory CNS disease	1 (0.8%)	2 (1.6%)	6 (1.3%)	8 (1.8%)
Thoracic vertebral canal stenosis associated with vertebral arch anomalies	0 (0.0%)	3 (2.4%)	0 (0.0%)	0 (0.0%)
Ischaemic myelopathy	0 (0.0%)	2 (1.6%)	0 (0.0%)	3 (0.7%)
Vertebral body osteomyelitis	0 (0.0%)	1 (0.8%)	0 (0.0%)	1 (0.2%)
Discospondylitis	0 (0.0%)	0 (0.0%)	1 (0.2%)	6 (1.3%)
Spinal epidural empyema	0 (0.0%)	0 (0.0%)	0 (0.0%)	1 (0.2%)
Spinal dysraphism	0 (0.0%)	0 (0.0%)	0 (0.0%)	1 (0.2%)
Total	125 (100.0%)		448 (100/0%)	

The group of 439 FBDs included in the study consisted of 292 males (192 neutered) and 147 females (105 neutered) aged between 3 and 120 months (median 44 months). The duration of clinical signs ranged from 1 to 639 days, and for cervical lesions, the neurological grade using the grading system described in the ‘Materials and Methods’ section included grade 4 (*n* = 55), grade 3 (*n* = 61) and grade 2 (*n* = 19). For thoracolumbar lesions, neurological grade using the modified Frankel score included grade 5 (*n* = 7), grade 4 (*n* = 115), grade 3 (*n* = 85), grade 2 (*n* = 61), grade 1 (*n* = 4) and grade 0 (*n* = 32). A total of 135 FBDs had a cervical lesion and 304 FBDs had a thoracolumbar lesion. Out of the 135 dogs with a cervical lesion, 122 (90.4%) were painful and 13 (9.6%) were non‐painful. Of the 304 dogs with a thoracolumbar lesion, 236 (77.6%) were painful and 68 (22.4%) were non‐painful. Seventy‐six (17.3%) FBDs had a peracute onset of clinical signs, 272 (62.0%) had an acute onset, 24 (5.5%) had a subacute onset and 67 (15.3%) had a chronic onset. Twenty‐four (5.5%) dogs had evidence of urinary or faecal incontinence, with five (1.1%) being faecally incontinent, eight (1.8%) being urinary incontinent and 11 (2.5%) being both urinary and faecally incontinent. The most common diagnosis for the FBDs was acute intervertebral disc extrusion (84.2% of total diagnoses, *n* = 377), followed by SAD (4.9% of total diagnoses, *n* = 22), inflammatory CNS disease (3.1% of total diagnoses, *n* = 14) and hemivertebrae with kyphosis (2.9% of total diagnoses, *n* = 13). Nine (2.1%) FBDs had multiple concurrent disease processes at the same or adjacent spinal cord segment.

The group of 106 pugs included in the study consisted of 70 males (35 neutered) and 36 females (20 neutered) aged between 3 and 180 months (median 71.5 months). The duration of clinical signs ranged from 1 to 1642 days, and for cervical lesions, neurological grade using the grading system described in the ‘Materials and Methods’ section included grade 4 (*n* = 2), grade 3 (*n* = 13) or grade 2 (*n* = 5). For thoracolumbar lesions, neurological grade using the modified Frankel score included grade 5 (*n* = 1), grade 4 (*n* = 75), grade 3 (*n* = 9) and grade 2 (*n* = 1). Twenty pugs had a cervical lesion and 86 pugs had a thoracolumbar lesion. Of the 20 pugs with a cervical lesion, nine (45.0%) were painful and 11 (55.0%) were non‐painful. Of the 86 pugs with a thoracolumbar lesion, 16 (18.6%) were painful and 70 (81.4%) were non‐painful. Of the 106 pugs in the study, nine (8.5%) had a peracute onset of clinical signs, 14 (13.2%) had an acute onset, four (3.8%) had a subacute onset and 79 (74.5%) had a chronic onset. Twenty‐two (20.8%) dogs had evidence of urinary or faecal incontinence, with nine (8.5%) being faecally incontinent, three (2.8%) being urinary incontinent and seven (6.6%) being both urinary and faecally incontinent. The two most common diagnoses in the pug group were SAD and chronic intervertebral disc protrusion, both with 24.0% of total diagnoses (*n* = 30), followed by hemivertebrae with kyphosis (16.0% of total diagnoses, *n* = 20) and constrictive myelopathy (12.0% of total diagnoses, *n* = 15). Nineteen (17.9%) pugs had multiple concurrent disease processes at the same or adjacent spinal cord segment.

**FIGURE 1:**
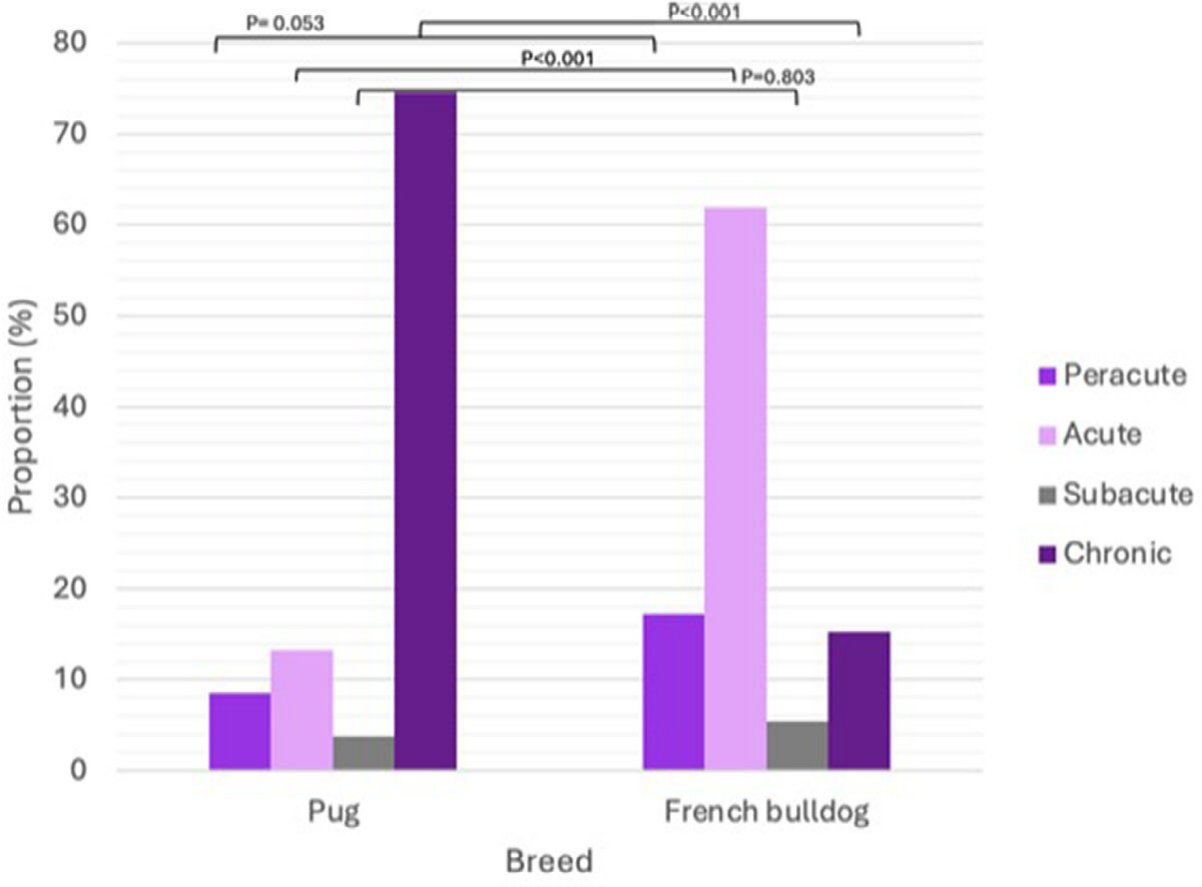
Bar graph showing the onset of clinical signs of spinal disease among pugs and French bulldogs

There was a significant difference in the onset of clinical signs between breeds, with FBDs being more likely to present with an acute onset of clinical signs and pugs being significantly more likely to have a chronic onset (*p* < 0.001 for both) (Figure 1). In addition, FBDs were significantly more likely to have a more severe neurological grade than pugs for both thoracolumbar (*p* < 0.001) and cervical (*p* = 0.027) lesions. FBDs presented with clinical signs of spinal disease at a significantly younger age than pugs (*p* < 0.01).

There was a significant difference in the distribution of diagnoses for spinal disease of FBDs and pugs (*p* < 0.001). A post hoc analysis was performed, excluding diagnoses with less than five cases, which revealed that this significant difference arose due to differences in the breed predisposition for acute intervertebral disc extrusion (*p* < 0.001), chronic intervertebral disc protrusion (*p* < 0.001), SAD (*p* < 0.001) and hemivertebrae with kyphosis (*p* < 0.001), with the former being more likely in FBDs and the latter three diagnoses being most likely in pugs. In addition, only nine (2.1%) FBDs had a concurrent disease process occurring at the same or adjacent spinal cord segment, whereas 19 (17.9%) pugs did (*p* < 0.001).

## DISCUSSION

The results of this study confirm our hypothesis that FBDs are most often affected by acute spinal disorders, such as acute intervertebral disc extrusions, whereas pugs seem to be more vulnerable to the development of chronic and complex spinal conditions, including chronic intervertebral disc protrusions, SADs and hemivertebrae with kyphosis. Despite their similar conformation, these breeds seem to be affected by a different spectrum of spinal disorders and consequently have different neurological presentations. For example, FBDs most commonly present with an acute onset of pain and neurological deficits, which are more severe than in pugs. In contrast, pugs typically have a chronic presentation of non‐painful neurological signs with a high prevalence of urinary or faecal incontinence. This study is in agreement with previous studies,^4^ which reported that 84.3% of FBDs with spinal disease were diagnosed with acute intervertebral disc extrusions. In contrast, the most common spinal disorders diagnosed in pugs in this study were SAD and chronic intervertebral disc protrusions, closely followed by hemivertebrae with kyphosis. The prevalence of spinal disease among pugs has not been reported previously, but existing literature has shown that they are overrepresented for SAD,^6^ meningeal fibrosis^19^ and hemivertebra with kyphosis.^10^

This study also highlights a difference in the prevalence of a cervical localisation among pugs and FBDs. Pugs had a lower incidence of cervical spinal cord disease compared to FBDs in this study, representing 16.0% and 30.5% of their respective populations. This can be explained by the most common diseases in each of the breeds. First, FBDs are most commonly affected by acute intervertebral disc extrusions, and a previous study has reported that 28.0% of these are in the cervical region,^30^ which is comparable to the findings of our study. In contrast, the diseases typically seen in this population of pugs are most often described in the thoracolumbar regions, including SAD and hemivertebrae with kyphosis.^5^, ^6^, ^10^, ^11^, ^31^

The exact reasons behind why both pugs and FBDs have a high prevalence of spinal disease are currently unknown. Conformational similarities leading to alterations in spinal biomechanics have been shown to lead to early onset degeneration,^32^, ^33^ which can explain the high prevalence of intervertebral disc disease among these two breeds.^4^ In addition, congenital vertebral malformations, which are frequent in both breeds,^10^, ^11^, ^13^, ^18^, ^22^ can lead to altered spinal biomechanics, which might contribute to the development of multiple spinal disorders, including constrictive myelopathy and SAD.^18^, ^19^, ^20^, ^31^, ^34^ It can further not be excluded that hypoxia associated with the brachycephalic phenotype can contribute to vertebral and spinal anomalies.^35^, ^36^, ^37^, ^38^

Despite their similar morphology, pugs and FBDs should not be considered closely related breeds,^39^ and existing literature has highlighted some clinically relevant differences in their neurological presentations. The existing knowledge about the distinction between spinal anatomy and genetics in pugs and FBDs may offer an explanation into the differences observed in this study regarding prevalence of different spinal diseases, and as a consequence, the onset of clinical signs.

FBDs have been reported to have a high incidence of 12‐FGF4 retrogene insertion, which has been shown to lead to an increased incidence of acute intervertebral disc extrusions and could contribute to the high prevalence found in this breed.^40^, ^41^, ^42^ Conversely, the reported allele frequency in pugs is very low, which may explain their relatively low frequency of acute intervertebral disc extrusions in this study. Dachshunds also have a high incidence of 12‐FGF4 insertion, potentially explaining their equally very high incidence of intervertebral disc disease. Despite the common genetics between dachshunds and FBDs, their phenotypes are vastly different, and pugs, which do not appear to share these genetics, are more similar in appearance to FBDs. In contrast to 12‐FGF4 insertion, 18‐FGF4 insertion has been shown to have only a minor effect on intervertebral disc disease but a more profound effect on body conformation.^40^ This mutation is commonly seen in the dachshund but not in the FBD, which may explain the conformational differences between the two breeds.

FBDs and pugs are both predisposed to the development of SADs,^6^ which are fluid‐filled dilatations of the subarachnoid space. In this study, it was the second most common disease process in FBDs and the most common in pugs, alongside chronic intervertebral disc protrusions. The prevalence of SADs in the population of FBDs in this study differs from previous studies, where a higher number have been reported.^4^ This may be as a consequence of excluding cases with disease at the same or adjacent site, leading to a falsely lower prevalence. It has been reported that concurrent disease processes at the same or adjacent site are common in both the pug and FBD,^5^, ^6^, ^43^ which has been reported in 3.3% and 61.5% of dogs, respectively.^6^ However, SADs have also been reported in the absence of concurrent disease, including in the cervical region of a population of young, related pugs,^8^ suggesting a genetic component of their development. CAPD has been hypothesised as a contributor to the development of SADs in the pug breed in particular.^20^ However, the relationship between SADs and vertebral malformations remains unclear due to their high prevalence in neurologically normal brachycephalic dogs.^10^, ^13^, ^16^ This is in contrast to other non‐brachycephalic breeds affected by SADs, which appear to have a low incidence of concurrent disease.^6^

In contrast to FBDs, in which acute intervertebral disc extrusions are the most common spinal disease,^4^ this study suggests that pugs are overrepresented for a different subtype of intervertebral disc disease: chronic intervertebral disc protrusions. Chronic intervertebral disc protrusions are more commonly seen in older, large‐breed non‐chondrodystrophic dogs,^44^ so it is perhaps unexpected that pugs, a small‐breed dog, seem to be predisposed. In addition, these lesions are often associated with complex multifactorial neurological diseases such as degenerative lumbosacral stenosis and disc‐associated cervical spondylomyelopathy,^45^, ^46^ two diseases that are not associated with the pug breed. However, pugs are known to suffer from a different form of multifactorial spinal disease, with multiple concurrent diagnoses contributing to their clinical signs.^34^, ^47^

In this study, clinically relevant hemivertebrae were the third most common diagnosis in pugs, whereas this condition was found to be uncommon in our population of FBDs. Vertebral malformations are common in both FBDs and pugs^10^, ^11^ with a 2018 study by Rohdin et al.^48^ reporting that 96% of pugs have at least one congenital vertebral malformation. Despite this, in many cases, they are found incidentally and not clearly causing neurological deficits.^10^, ^15^, ^16^ While the two breeds are phenotypically similar, many differences in their spinal anatomy have already been described, particularly regarding the presence and characteristics of vertebral malformations. For example, pugs have been found to suffer from a different subtype of hemivertebrae than FBDs^13^, ^16^ and are also more likely to be affected by clinically relevant vertebral body malformations.^10^ Extrapolating information from previous literature may provide an explanation for the differences seen in the prevalence of this condition between the two breeds studied. Previous studies have indeed demonstrated a different prevalence, anatomical characteristics and clinical relevance of hemivertebra between FBDs and pugs.^14^, ^16^ Hemivertebra in FBDs have been associated with the screw‐tail morphology^14^, ^49^ and a variant in the DISHEVELLED 2 gene.^14^ Although the aetiology of hemivertebra in pugs is currently unclear, they should not be considered screw‐tailed brachycephalic dogs and they do not display the aforementioned genetic variant.^14^

Thoracolumbar myelopathies in pugs present a frequent diagnostic challenge. It is common to find multiple spinal cord disorders concurrently in pugs,^34^ including constrictive myelopathy, SAD and chronic intervertebral disc protrusions. Additionally, it is possible that pugs diagnosed with a single disease process in this study may have had concurrent meningeal fibrosis, which is a histopathological diagnosis, without imaging evidence.^19^ In this study, 17.9% of pugs had more than one diagnosis at the same or adjacent spinal cord segment. This can present a clinical challenge when diagnosing and managing pugs with spinal disease. The occurrence of chronic progressive ataxia and paresis of the pelvic limbs in pugs has been referred to as ‘pug dog thoracolumbar myelopathy’.^34^, ^47^ The aetiology of thoracolumbar myelopathy in pugs is complex and possibly multifactorial. A recent study by Brander et al.^47^ has identified several candidate genes that may be responsible for the complexity of the abnormalities seen in pugs with thoracolumbar myelopathies.

This study is limited by its retrospective nature, meaning that there was no standardised diagnostic protocol for the animals included in the study. As such, it is possible that some relevant spinal lesions were not observed due to the differences in the MRI protocol followed. In addition, some patients also underwent a post‐MRI CT scan. Imaging studies did not undergo re‐evaluation for the purpose of this study, which may have had an effect on disease prevalence due to changes in knowledge of MRI features over the course of the study period. Patients with more than one concurrent disease process that was not at the same or adjacent spinal cord segment were excluded from the study, which could have led to a falsely lowered prevalence of some diagnoses. In particular, this may have affected the pug population, who are well known to present with multiple lesions. For those with more than one diagnosis at the same or adjacent site, it was not possible to conclude whether the clinical signs were caused by one specified lesion or both in combination.^19^, ^20^

This study highlights clear differences between the presentation and diagnoses of pugs and FBDs with clinical signs of spinal disease. This complements our understanding of the variations in vertebral anatomy and genetics between the two breeds, which has already been highlighted in existing literature. Altogether, this emphasises the importance of recognising these two phenotypically similar breeds separately in order to consider the most likely differential diagnoses, the most appropriate diagnostic tests and treatment options and ultimately the likely prognosis.

## AUTHOR CONTRIBUTIONS

Stephanie Kerr, Lowri Maskell and Steven De Decker conceived the study and participated in its design. Stephanie Kerr and Steven De Decker wrote the final version of the manuscript. Stephanie Kerr, Eleftheria Skovola, Lowri Maskell and Steven De Decker collected, analysed and interpreted the data. All the authors have read and approved the final manuscript.

## CONFLICT OF INTEREST STATEMENT

The authors declare they have no conflicts of interest.

## FUNDING INFORMATION

The study received no specific funding for this study.

## ETHICS STATEMENT

Formal ethical approval was not required for this study due to its retrospective nature using data from electronic medical records database at the RVC, which is in line with GDPR regulations.

## Data Availability

The data are available from the corresponding author upon reasonable request.
